# Dicarbon­yl[4-(2,6-dimethyl­phenyl­amino)­pent-3-en-2-onato-κ^2^ *N*,*O*]rhodium(I)

**DOI:** 10.1107/S1600536812017175

**Published:** 2012-04-25

**Authors:** Gertruida J. S. Venter, Gideon Steyl, Andreas Roodt

**Affiliations:** a Department of Chemistry, University of the Free State, PO Box 339, Bloemfontein 9300, South Africa

## Abstract

In the title compound, [Rh(C_13_H_16_NO)(CO)_2_], a square-planar coordination geometry is observed around the Rh^I^ atom, formed by the N and O atoms of the bidentate ligand and two C atoms from two carbonyl ligands. The Rh^I^ atom is displaced from the plane through these surrounding atoms by 0.0085 (2) Å. The dihedral angle between the benzene ring and the N—C—C—C—O plane is 89.82 (6)°, and the N—Rh—O bite angle for the bidentate ligand is 90.53 (6)°. An inter­molecular C—H⋯O inter­action is observed between a methyl group of the benzene ring and a carbonyl O atom.

## Related literature

For background to the ligand preparation, see: Shaheen *et al.* (2006 ▶); Venter *et al.* (2010*a* ▶,*b* ▶). For applications of rhodium compounds containing bidentate ligand systems, see: Cornils & Herrmann (1996 ▶); Steyn *et al.* (1997 ▶); Trzeciak & Ziolkowski (1994 ▶); Van Rooy *et al.* (1995 ▶). For related rhodium enamino­ketonato complexes, see: Brink *et al.* (2010 ▶); Damoense *et al.* (1994 ▶); Otto *et al.* (1998 ▶); Roodt *et al.* (2011 ▶); Steyn *et al.* (1992 ▶); Varshavsky *et al.* (2001 ▶); Venter *et al.* (2009*a* ▶,*b* ▶).
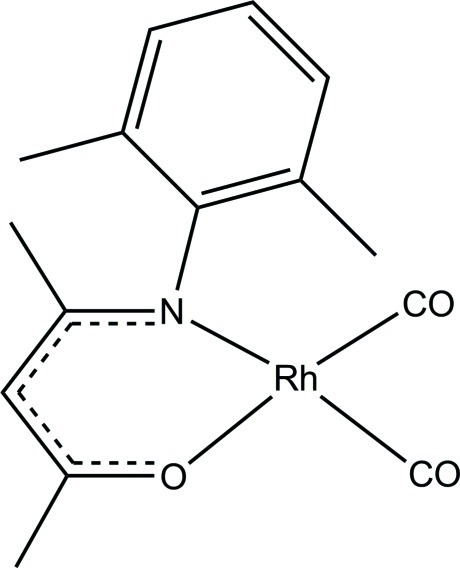


## Experimental

### 

#### Crystal data

[Rh(C_13_H_16_NO)(CO)_2_]
*M* *_r_* = 361.2
Orthorhombic, 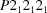

*a* = 7.9191 (7) Å
*b* = 12.3873 (5) Å
*c* = 15.393 (6) Å
*V* = 1510.0 (6) Å^3^
*Z* = 4
Mo *K*α radiation
μ = 1.14 mm^−1^
*T* = 100 K
0.34 × 0.20 × 0.11 mm

#### Data collection

Bruker APEXII CCD diffractometer
Absorption correction: multi-scan (*SADABS*; Bruker, 2001 ▶) *T*_min_ = 0.699, *T*_max_ = 0.885
20802 measured reflections
3767 independent reflections
3746 reflections with *I* > 2σ(*I*)
*R*_int_ = 0.029

#### Refinement

*R*[*F* ^2^ > 2σ(*F* ^2^)] = 0.017
*wR*(*F* ^2^) = 0.042
*S* = 1.07
3767 reflections
174 parameters
H-atom parameters constrained
Δρ_max_ = 0.79 e Å^−3^
Δρ_min_ = −0.57 e Å^−3^
Absolute structure: Flack (1983 ▶), 1605 Friedel pairs
Flack parameter: −0.01 (2)

### 

Data collection: *APEX2* (Bruker, 2007 ▶); cell refinement: *SAINT-Plus* (Bruker, 2007 ▶); data reduction: *SAINT-Plus*; program(s) used to solve structure: *SHELXS97* (Sheldrick, 2008 ▶); program(s) used to refine structure: *SHELXL97* (Sheldrick, 2008 ▶); molecular graphics: *DIAMOND* (Brandenburg & Putz, 1999 ▶); software used to prepare material for publication: *WinGX* (Farrugia, 1999 ▶).

## Supplementary Material

Crystal structure: contains datablock(s) global, I. DOI: 10.1107/S1600536812017175/hy2537sup1.cif

Structure factors: contains datablock(s) I. DOI: 10.1107/S1600536812017175/hy2537Isup2.hkl

Additional supplementary materials:  crystallographic information; 3D view; checkCIF report

## Figures and Tables

**Table 1:** Hydrogen-bond geometry (Å, °)

*D*—H⋯*A*	*D*—H	H⋯*A*	*D*⋯*A*	*D*—H⋯*A*
C118—H11*D*⋯O12^i^	0.98	2.51	3.441 (2)	159

Symmetry code: (i) 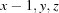.

**Table 2:** Comparative geometrical parameters for the title complex with [Rh(*N*,*O*-bid)(CO)(PPh_3_)]*^*a*^* complexes (Å, °)

Parameters	I	II	III	IV	V
Rh1—N11	2.048 (2)	2.077 (2)	2.069 (2)	2.045 (4)	2.045 (3)
Rh1—O12	2.021 (1)	2.027 (2)	2.028 (2)	2.044 (3)	2.045 (2)
Rh1—C13*^*b*^*	1.880 (2)	2.2704 (7)	2.2635 (6)	2.275 (1)	2.281 (2)
Rh1—C14	1.852 (2)	1.812 (3)	1.807 (2)	1.784 (5)	1.804 (3)
N11⋯O12	2.890 (2)	2.885 (3)	2.885 (3)	2.826 (6)	2.841 (3)
N11—Rh1—O12	90.53 (6)	89.31 (9)	89.54 (8)	87.4 (1)	87.95 (8)
O12—Rh1—C13*^*b*^*	87.25 (8)	85.95 (6)	84.97 (5)	89.7 (1)	89.91 (5)
C13*^*b*^*—Rh1—C14	89.8 (1)	91.57 (9)	91.87 (7)	90.3 (2)	89.48 (9)
N11—Rh1—C14	92.41 (8)	93.1 (1)	93.6 (1)	92.6 (2)	92.6 (1)
N11—C2—C4—O12	1.6 (2)	−2.6 (2)	4.1 (2)	1.2 (4)	1.5 (2)

(I) This work. (II) Carbon­yl[4-(2,6-dimethyl­phenyl­amino)­pent-3-en-2-onato- κ^2^ *N*,*O*](triphenyl­phosphine-κ*P*)rhodium(I) (Venter *et al.*, 2009*b* ▶). (III) Carbon­yl[4-(2,3-dimeth­yl­phenyl­amino)­pent-3-en-2-onato-κ^2^ *N*,*O*](triphenyl­phosphine-κ*P*)rhodium(I) (Venter *et al.*, 2009*a* ▶). (IV) Carbon­yl(4-amino­pent-3-en-2-onato-κ^2^ *N*,*O*)(triphenyl­phosphine-κ*P*)rhodium(I) (Damoense *et al.*, 1994 ▶). (V) Carbon­yl(4-amino-1,1,1-trifluoro-pent-3-en-2-onato-κ^2^ *N*,*O*)(triphenyl­phosphine-κ*P*)rhodium(I) (Varshavsky *et al.*, 2001 ▶). (*a*) *N*,*O*-bid is a mono-anionic bidentate ligand coordinated to a metal *via* (*N*,*O*) donor atoms. (*b*) P13 atom is used in comparative complexes instead of C13 atom.

**Fig. 1.**
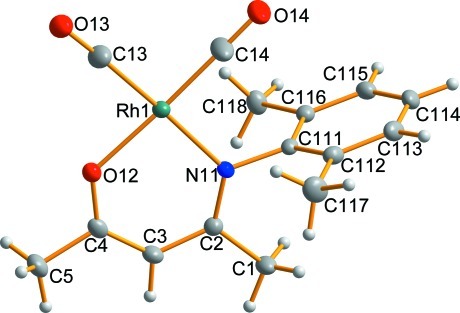
Molecular structure of the title compound. Displacement ellipsoids are drawn at the 50% probability level.

## supplementary crystallographic information

### Comment

Rhodium(I) dicarbonyl complexes of the type [Rh(*L*,*L*')(CO)_2_], where *L*,*L*' is a chelating mono-anionic bidentate ligand coordinated to rhodium *via* (O, O') donor atoms, have been studied as catalyst precursors (Cornils & Herrmann, 1996; Trzeciak & Ziolkowski, 1994; Van Rooy *et al.*, 1995). In this study the investigation of these complexes is followed by complexes containing bidentate β-enaminoketonato ligands such as 4-(phenylamino)pent-3-en-2-onato (Phony) (Shaheen *et al.*, 2006; Venter *et al.*, 2010*a*,*b*) coordinated to rhodium *via* (N, O) donor atoms. Studies have also been conducted involving complexes of the type [Rh(*L*,*L*')(CO)(PPh_3_)], where one of the CO-ligands in the [Rh(*L*,*L*')(CO)_2_] complex is substituted by a PPh_3_ ligand (Brink *et al.*, 2010; Otto *et al.*, 1998; Roodt *et al.*, 2011; Steyn *et al.*, 1992), as well as the mechanism of oxidative addition of methyl iodide to complexes of this type (Steyn *et al.*, 1997).

Bond distances involving the Rh^I^ atom in the title complex differ from complexes in literature involving triphenylphosphine as ligand, with especially the Rh1—C14 distance that is significantly longer (Table 2). The title complex displays similar Rh—N distances to complexes containing a hydrogen atom instead of an aryl moiety, but is more closely related to complexes containing similar aryl moieties when comparing the Rh—O distance. Complexes containing a bulky substituent on the nitrogen atom also display larger N—Rh—O bite angles than complexes containing a hydrogen atom. Due to the *trans* influence of the nitrogen atom, the Rh1—C13 distance is significantly longer than the Rh1—C14 distance [1.880 (2) and 1.852 (2) Å].

Intermolecular C—H···O interaction is observed between a methyl group on the aryl moiety and oxygen of the bidentate ligand. The dihedral angle between the plane formed by the N, O and C atoms of the pentenone backbone and the aryl ring moiety is 89.82 (6) °. This angle is distorted from the ideal value of 0° for delocalized electron due to the steric interference of the aryl ring, and is exacerbated by the presence of the methyl groups on the ring.

### Experimental

[RhCl(CO)_2_]_2_ was prepared *in situ* by heating RhCl_3_.3H_2_O (0.1014 g, 0.385 mmol) in 2 ml DMF under reflux for 30 min. 2,6-diMe-PhonyH (0.0892 g, 0.439 mmol) was added to the cooled DMF solution of [RhCl(CO)_2_]_2_. The product was precipitated by ice-water and centrifuge, and recrystallized from acetone. Yellow crystals suitable for X-Ray diffraction were collected in 80.09% yield (0.1114 g). IR (KBr, cm^-1^): ν_CO, _*_sym_* 2064.5 s, ν_CO, _*_asym_* 1985.8 s.

### Refinement

H atoms were placed in geometrically idealized positions and constrained to ride on their parent atoms, with C—H = 0.95 (aromatic) and 0.98 (methyl) Å and *U*_iso_(H) = 1.2(1.5 for methyl)*U*_eq_(C).

### Crystal data

**Table:**

[Rh(C_13_H_16_NO)(CO)_2_]	*F*(000) = 728
*M**_r_* = 361.2	*D*_x_ = 1.589 Mg m^−^^3^
Orthorhombic, *P*2_1_2_1_2_1_	Mo *K*α radiation, λ = 0.71073 Å
Hall symbol: P 2ac 2ab	Cell parameters from 9513 reflections
*a* = 7.9191 (7) Å	θ = 2.9–28.3°
*b* = 12.3873 (5) Å	µ = 1.14 mm^−^^1^
*c* = 15.393 (6) Å	*T* = 100 K
*V* = 1510.0 (6) Å^3^	Cuboid, yellow
*Z* = 4	0.34 × 0.20 × 0.11 mm

### Data collection

**Table:**

Bruker APEXII CCD diffractometer	3767 independent reflections
Radiation source: fine-focus sealed tube	3746 reflections with *I* > 2σ(*I*)
Graphite monochromator	*R*_int_ = 0.029
φ and ω scans	θ_max_ = 28.3°, θ_min_ = 2.7°
Absorption correction: multi-scan (*SADABS*; Bruker, 2001)	*h* = −9→10
*T*_min_ = 0.699, *T*_max_ = 0.885	*k* = −16→16
20802 measured reflections	*l* = −17→20

### Refinement

**Table:**

Refinement on *F*^2^	Hydrogen site location: inferred from neighbouring sites
Least-squares matrix: full	H-atom parameters constrained
*R*[*F*^2^ > 2σ(*F*^2^)] = 0.017	*w* = 1/[σ^2^(*F*_o_^2^) + (0.0159*P*)^2^ + 0.9823*P*] where *P* = (*F*_o_^2^ + 2*F*_c_^2^)/3
*wR*(*F*^2^) = 0.042	(Δ/σ)_max_ = 0.002
*S* = 1.07	Δρ_max_ = 0.79 e Å^−^^3^
3767 reflections	Δρ_min_ = −0.57 e Å^−^^3^
174 parameters	Extinction correction: *SHELXL97* (Sheldrick, 2008)
0 restraints	Extinction coefficient: 0.0163 (5)
Primary atom site location: structure-invariant direct methods	Absolute structure: Flack (1983), 1605 Friedel pairs
Secondary atom site location: difference Fourier map	Flack parameter: −0.01 (2)

### Special details

**Table:**

Experimental. The intensity data was collected on a Bruker X8 APEXII 4 K Kappa CCD diffractometer using an exposure time of 60 s/frame. A total of 1033 frames were collected with a frame width of 0.5° covering up to θ = 28.31° with 99.8% completeness accomplished.
Geometry. All s.u.'s (except the s.u. in the dihedral angle between two l.s. planes) are estimated using the full covariance matrix. The cell s.u.'s are taken into account individually in the estimation of s.u.'s in distances, angles and torsion angles; correlations between s.u.'s in cell parameters are only used when they are defined by crystal symmetry. An approximate (isotropic) treatment of cell s.u.'s is used for estimating s.u.'s involving l.s. planes.
Refinement. Refinement of *F*^2^ against ALL reflections. The weighted *R*-factor *wR* and goodness of fit *S* are based on *F*^2^, conventional *R*-factors *R* are based on *F*, with *F* set to zero for negative *F*^2^. The threshold expression of *F*^2^ > 2σ(*F*^2^) is used only for calculating *R*-factors(gt) *etc*. and is not relevant to the choice of reflections for refinement. *R*-factors based on *F*^2^ are statistically about twice as large as those based on *F*, and *R*- factors based on ALL data will be even larger.

### Fractional atomic coordinates and isotropic or equivalent isotropic displacement parameters (Å^2^)

**Table:**

	*x*	*y*	*z*	*U*_iso_*/*U*_eq_	
C1	−0.1665 (3)	0.74776 (15)	0.96601 (14)	0.0256 (4)	
H1A	−0.2853	0.7323	0.9532	0.038*	
H1B	−0.1389	0.821	0.9465	0.038*	
H1C	−0.1472	0.7421	1.0287	0.038*	
C2	−0.0554 (2)	0.66732 (14)	0.91901 (11)	0.0172 (3)	
C3	0.0594 (3)	0.71073 (13)	0.85745 (11)	0.0192 (3)	
H3	0.0598	0.787	0.8512	0.023*	
C4	0.1704 (2)	0.65405 (15)	0.80574 (12)	0.0182 (3)	
C5	0.2854 (3)	0.71425 (17)	0.74432 (13)	0.0261 (4)	
H5A	0.4033	0.7006	0.7602	0.039*	
H5B	0.2621	0.7918	0.7481	0.039*	
H5C	0.2654	0.6894	0.6848	0.039*	
C13	0.1968 (3)	0.33435 (18)	0.82539 (16)	0.0315 (3)	
C14	−0.0362 (4)	0.33708 (18)	0.94754 (18)	0.0431 (4)	
C111	−0.2003 (2)	0.53224 (12)	0.99790 (11)	0.0155 (3)	
C112	−0.1618 (3)	0.52091 (15)	1.08635 (12)	0.0202 (4)	
C113	−0.2906 (3)	0.48830 (15)	1.14235 (12)	0.0247 (4)	
H113	−0.2669	0.4799	1.2025	0.03*	
C114	−0.4521 (3)	0.46795 (14)	1.11235 (13)	0.0251 (4)	
H114	−0.5385	0.4473	1.1518	0.03*	
C115	−0.4876 (2)	0.47775 (14)	1.02404 (13)	0.0214 (4)	
H115	−0.598	0.4624	1.0033	0.026*	
C116	−0.3627 (2)	0.50984 (14)	0.96590 (12)	0.0169 (3)	
C117	0.0141 (3)	0.54302 (18)	1.11936 (14)	0.0295 (4)	
H11A	0.023	0.5194	1.18	0.044*	
H11B	0.0375	0.6206	1.1157	0.044*	
H11C	0.096	0.5034	1.0839	0.044*	
C118	−0.3997 (2)	0.51770 (16)	0.87001 (12)	0.0229 (4)	
H11D	−0.5215	0.5101	0.8604	0.034*	
H11E	−0.3397	0.4601	0.8391	0.034*	
H11F	−0.362	0.588	0.8482	0.034*	
N11	−0.0694 (2)	0.56317 (11)	0.93686 (9)	0.0153 (2)	
O12	0.18814 (16)	0.55013 (11)	0.80425 (8)	0.0188 (2)	
O13	0.2774 (2)	0.27212 (12)	0.79070 (11)	0.0315 (3)	
O14	−0.0990 (3)	0.27211 (12)	0.98856 (13)	0.0431 (4)	
Rh1	0.066650 (17)	0.441740 (10)	0.879887 (8)	0.01597 (5)	

### Atomic displacement parameters (Å^2^)

**Table:**

	*U*^11^	*U*^22^	*U*^33^	*U*^12^	*U*^13^	*U*^23^
C1	0.0296 (10)	0.0145 (8)	0.0327 (10)	0.0009 (8)	0.0099 (8)	0.0002 (7)
C2	0.0171 (8)	0.0171 (7)	0.0172 (7)	0.0007 (7)	−0.0010 (7)	−0.0001 (6)
C3	0.0205 (8)	0.0153 (7)	0.0219 (8)	−0.0015 (7)	−0.0008 (8)	0.0029 (6)
C4	0.0163 (8)	0.0216 (8)	0.0168 (8)	−0.0042 (7)	−0.0036 (6)	0.0030 (6)
C5	0.0250 (10)	0.0264 (9)	0.0268 (10)	−0.0043 (8)	0.0051 (8)	0.0059 (8)
C13	0.0282 (6)	0.0244 (6)	0.0419 (7)	0.0026 (5)	0.0101 (5)	−0.0036 (5)
C14	0.0567 (10)	0.0183 (5)	0.0543 (8)	0.0045 (6)	0.0297 (8)	0.0040 (5)
C111	0.0197 (8)	0.0119 (7)	0.0150 (7)	0.0013 (6)	0.0022 (6)	0.0002 (5)
C112	0.0263 (10)	0.0169 (8)	0.0175 (8)	−0.0014 (7)	−0.0018 (7)	0.0000 (6)
C113	0.0384 (12)	0.0210 (8)	0.0147 (8)	−0.0045 (8)	0.0017 (8)	0.0010 (6)
C114	0.0343 (10)	0.0196 (8)	0.0215 (8)	−0.0060 (7)	0.0098 (9)	0.0005 (6)
C115	0.0211 (9)	0.0168 (8)	0.0263 (9)	−0.0024 (7)	0.0038 (7)	−0.0015 (7)
C116	0.0193 (8)	0.0138 (7)	0.0177 (8)	0.0006 (6)	0.0010 (7)	0.0006 (6)
C117	0.0315 (9)	0.0357 (10)	0.0213 (8)	−0.0033 (8)	−0.0078 (8)	0.0027 (9)
C118	0.0196 (8)	0.0301 (9)	0.0191 (9)	−0.0017 (7)	−0.0023 (7)	0.0030 (7)
N11	0.0160 (6)	0.0149 (6)	0.0150 (6)	−0.0030 (7)	0.0004 (6)	0.0005 (5)
O12	0.0171 (6)	0.0194 (6)	0.0198 (6)	−0.0009 (6)	0.0010 (5)	0.0001 (5)
O13	0.0282 (6)	0.0244 (6)	0.0419 (7)	0.0026 (5)	0.0101 (5)	−0.0036 (5)
O14	0.0567 (10)	0.0183 (5)	0.0543 (8)	0.0045 (6)	0.0297 (8)	0.0040 (5)
Rh1	0.01566 (6)	0.01375 (6)	0.01848 (7)	0.00045 (5)	0.00126 (5)	−0.00015 (5)

### Geometric parameters (Å, º)

**Table:**

C1—C2	1.514 (3)	C111—N11	1.451 (2)
C1—H1A	0.98	C112—C113	1.395 (3)
C1—H1B	0.98	C112—C117	1.508 (3)
C1—H1C	0.98	C113—C114	1.383 (3)
C2—N11	1.324 (2)	C113—H113	0.95
C2—C3	1.419 (3)	C114—C115	1.393 (3)
C3—C4	1.378 (3)	C114—H114	0.95
C3—H3	0.95	C115—C116	1.392 (3)
C4—O12	1.295 (2)	C115—H115	0.95
C4—C5	1.510 (3)	C116—C118	1.508 (3)
C5—H5A	0.98	C117—H11A	0.98
C5—H5B	0.98	C117—H11B	0.98
C5—H5C	0.98	C117—H11C	0.98
C13—O13	1.134 (3)	C118—H11D	0.98
C13—Rh1	1.880 (2)	C118—H11E	0.98
C14—O14	1.138 (3)	C118—H11F	0.98
C14—Rh1	1.852 (2)	N11—Rh1	2.0476 (15)
C111—C112	1.402 (3)	O12—Rh1	2.0209 (13)
C111—C116	1.405 (3)		
			
C2—C1—H1A	109.5	C113—C114—C115	119.77 (18)
C2—C1—H1B	109.5	C113—C114—H114	120.1
H1A—C1—H1B	109.5	C115—C114—H114	120.1
C2—C1—H1C	109.5	C116—C115—C114	120.58 (19)
H1A—C1—H1C	109.5	C116—C115—H115	119.7
H1B—C1—H1C	109.5	C114—C115—H115	119.7
N11—C2—C3	124.18 (16)	C115—C116—C111	118.78 (17)
N11—C2—C1	119.57 (16)	C115—C116—C118	120.65 (17)
C3—C2—C1	116.24 (15)	C111—C116—C118	120.55 (16)
C4—C3—C2	126.95 (16)	C112—C117—H11A	109.5
C4—C3—H3	116.5	C112—C117—H11B	109.5
C2—C3—H3	116.5	H11A—C117—H11B	109.5
O12—C4—C3	125.86 (17)	C112—C117—H11C	109.5
O12—C4—C5	114.49 (17)	H11A—C117—H11C	109.5
C3—C4—C5	119.65 (17)	H11B—C117—H11C	109.5
C4—C5—H5A	109.5	C116—C118—H11D	109.5
C4—C5—H5B	109.5	C116—C118—H11E	109.5
H5A—C5—H5B	109.5	H11D—C118—H11E	109.5
C4—C5—H5C	109.5	C116—C118—H11F	109.5
H5A—C5—H5C	109.5	H11D—C118—H11F	109.5
H5B—C5—H5C	109.5	H11E—C118—H11F	109.5
O13—C13—Rh1	177.7 (2)	C2—N11—C111	116.87 (15)
O14—C14—Rh1	179.4 (2)	C2—N11—Rh1	125.65 (12)
C112—C111—C116	121.30 (17)	C111—N11—Rh1	117.35 (10)
C112—C111—N11	120.00 (17)	C4—O12—Rh1	126.77 (12)
C116—C111—N11	118.64 (15)	C14—Rh1—C13	89.80 (11)
C113—C112—C111	118.04 (18)	C14—Rh1—O12	176.97 (9)
C113—C112—C117	121.31 (17)	C13—Rh1—O12	87.25 (8)
C111—C112—C117	120.65 (18)	C14—Rh1—N11	92.41 (8)
C114—C113—C112	121.50 (18)	C13—Rh1—N11	177.76 (9)
C114—C113—H113	119.2	O12—Rh1—N11	90.53 (6)
C112—C113—H113	119.2		
			
N11—C2—C3—C4	1.4 (3)	N11—C111—C116—C118	0.1 (2)
C1—C2—C3—C4	−178.66 (18)	C3—C2—N11—C111	−176.47 (16)
C2—C3—C4—O12	0.4 (3)	C1—C2—N11—C111	3.6 (2)
C2—C3—C4—C5	−179.26 (18)	C3—C2—N11—Rh1	−0.8 (3)
C116—C111—C112—C113	−1.1 (3)	C1—C2—N11—Rh1	179.31 (13)
N11—C111—C112—C113	−178.36 (15)	C112—C111—N11—C2	−94.1 (2)
C116—C111—C112—C117	178.70 (17)	C116—C111—N11—C2	88.55 (19)
N11—C111—C112—C117	1.4 (3)	C112—C111—N11—Rh1	89.86 (16)
C111—C112—C113—C114	−0.3 (3)	C116—C111—N11—Rh1	−87.51 (17)
C117—C112—C113—C114	180.00 (18)	C3—C4—O12—Rh1	−2.7 (3)
C112—C113—C114—C115	1.4 (3)	C5—C4—O12—Rh1	177.03 (12)
C113—C114—C115—C116	−1.2 (3)	C4—O12—Rh1—C13	−177.21 (16)
C114—C115—C116—C111	−0.1 (3)	C4—O12—Rh1—N11	2.46 (14)
C114—C115—C116—C118	178.41 (17)	C2—N11—Rh1—C14	178.50 (18)
C112—C111—C116—C115	1.2 (3)	C111—N11—Rh1—C14	−5.83 (15)
N11—C111—C116—C115	178.54 (15)	C2—N11—Rh1—O12	−0.78 (15)
C112—C111—C116—C118	−177.26 (17)	C111—N11—Rh1—O12	174.89 (12)

### Hydrogen-bond geometry (Å, º)

**Table:**

*D*—H···*A*	*D*—H	H···*A*	*D*···*A*	*D*—H···*A*
C118—H11*D*···O12^i^	0.98	2.51	3.441 (2)	159

Symmetry code: (i) *x*−1, *y*, *z*.

### Comparative geometrical parameters for the title complex with [Rh(N,O-bid)(CO)(PPh_3_)]*^a^* complexes (Å, °).

**Table:**

Parameters	I	II	III	IV	V
Rh1—N11	2.048 (2)	2.077 (2)	2.069 (2)	2.045 (4)	2.045 (3)
Rh1—O12	2.021 (1)	2.027 (2)	2.028 (2)	2.044 (3)	2.045 (2)
Rh1—C13*^b^*	1.880 (2)	2.2704 (7)	2.2635 (6)	2.275 (1)	2.281 (2)
Rh1—C14	1.852 (2)	1.812 (3)	1.807 (2)	1.784 (5)	1.804 (3)
N11···O12	2.890 (2)	2.885 (3)	2.885 (3)	2.826 (6)	2.841 (3)
N11—Rh1—O12	90.53 (6)	89.31 (9)	89.54 (8)	87.4 (1)	87.95 (8)
O12—Rh1—C13*^b^*	87.25 (8)	85.95 (6)	84.97 (5)	89.7 (1)	89.91 (5)
C13*^b^*—Rh1—C14	89.8 (1)	91.57 (9)	91.87 (7)	90.3 (2)	89.48 (9)
N11—Rh1—C14	92.41 (8)	93.1 (1)	93.6 (1)	92.6 (2)	92.6 (1)
N11—C2—C4—O12	1.6 (2)	-2.6 (2)	4.1 (2)	1.2 (4)	1.5 (2)

(I) This work. (II) Carbonyl[4-(2,6-dimethylphenylamino)pent-3-en-2-onato- κ^2^*N*,*O*](triphenylphosphine-κ*P*)rhodium(I) (Venter *et al.*, 2009*b*). (III) Carbonyl[4-(2,3-dimethylphenylamino)pent -3-en-2-onato-κ^2^*N*,*O*](triphenylphosphine-κ*P*)rhodium(I) (Venter *et al.*, 2009*a*). (IV) Carbonyl(4-aminopent-3-en-2-onato-κ^2^*N*,*O*) (triphenylphosphine-κ*P*)rhodium(I) (Damoense *et al.*, 1994). (V) Carbonyl(4-amino-1,1,1-trifluoro-pent-3-en-2-onato-κ^2^*N*,*O*) (triphenylphosphine-κ*P*)rhodium(I) (Varshavsky *et al.*, 2001). (a) N,O-bid is a mono-anionic bidentate ligand coordinated to a metal *via* (*N*,*O*) donor atoms. (b) P13 atom is used in comparative complexes instead of C13 atom.
